# Supplementary material for: Rapid poxvirus engineering using CRISPR/Cas9 as a selection tool
Source: Commun Biol. 2020 Nov 3;3:643. doi: 10.1038/s42003-020-01374-6 (PMC7641209; doi:10.1038/s42003-020-01374-6)
Supplement: Supplementary file 1 — Supplementary Information [file 42003_2020_1374_MOESM1_ESM.pdf]

SUPPLEMENTARY INFORMATION

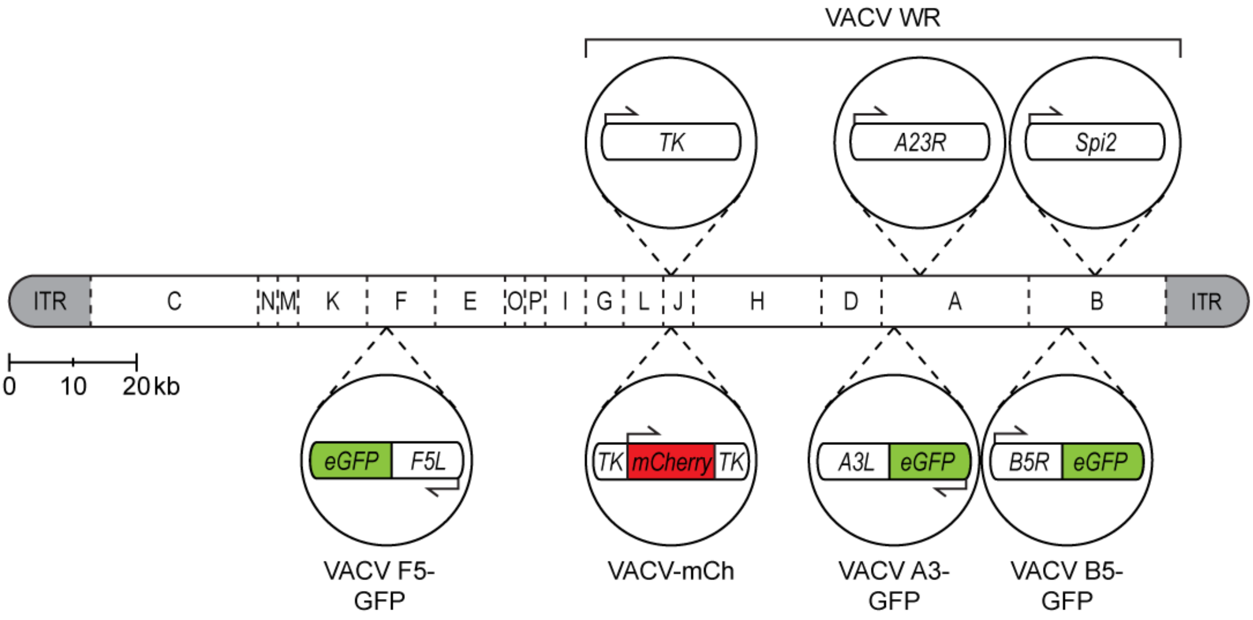

Supplementary Figure 1 – VACVs used in this study and targeted gene loci. VACV genome marked with *HindIII* fragments and position of inverted terminal repeat (ITR) regions. Locations of Cas9/gRNA-targeted genes are in circles that are labelled with the names of corresponding viruses (circles).

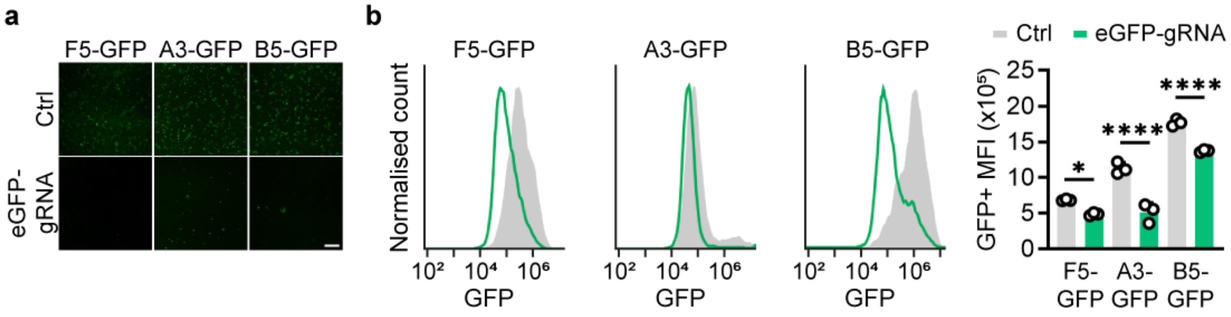

Supplementary Figure 2 – GFP expression is reduced by Cas9 regardless of VACV genome location. a. Fluorescence micrographs of cells infected with VACV F5-GFP, A3-GFP and B5-GFP at 48 hpi and transfected with Cas9/eGFP-gRNA. **b.** Representative histograms of flow cytometry data and MFI of GFP^+ cells are also shown. Graph represents three replicate cultures and depicts mean and SEM (*p<0.05, ****p<0.0001 by one-way ANOVA).

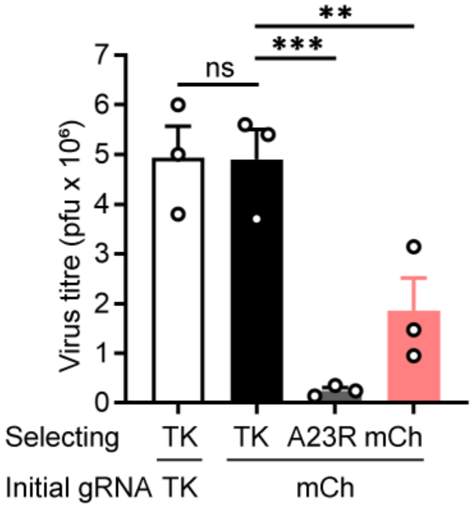

Supplementary Figure 3 – Selection efficacy correlates with virus titre. Titrations of progeny virus from selecting Cas9/gRNA cultures from experiments shown in Fig. 5a with gRNAs in initial and selecting cultures shown. Graphs show means and SEM across three independent experiments (ns p>0.05, * p<0.05, **p<0.01 by two-way ANOVA).

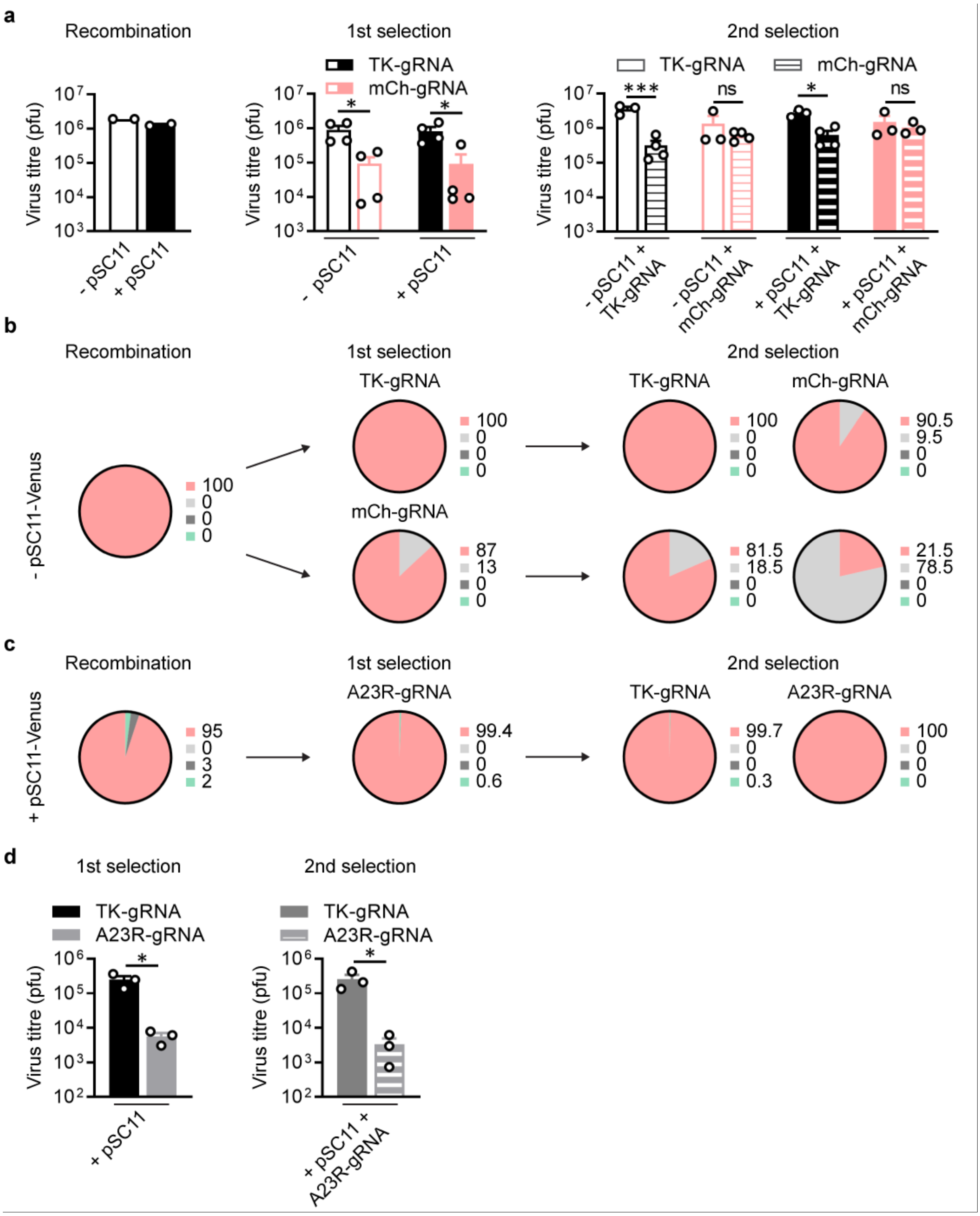

Supplementary Figure 4 – Success of selection is reflected in virus titres. a. Titres of progeny from each step shown in Figure 6a (+pSC11) and where the same protocol was followed but there were no recombinants generated in the first step (-pSC11). **b**. The protocol shown in Figure 6a was followed except no recombination template was provided in the first step. **c, d.** Progeny of initial recombinations shown in Figure 6b (reproduced as the pie under "Recombination") were grown on selecting cultures transfected with Cas9/A23R-gRNA before harvesting and

screening for fluorescence (**c**) and total plaque counts (**d)**. Pie charts and adjacent numbers show results of 100 plaques screened for fluorescence in each condition and across two independent experiments. Graphs depict means and SEM of virus titres (ns $p>0.05$, * $p<0.05$, ** $p<0.01$, **** $p<0.0001$ by one-way ANOVA).

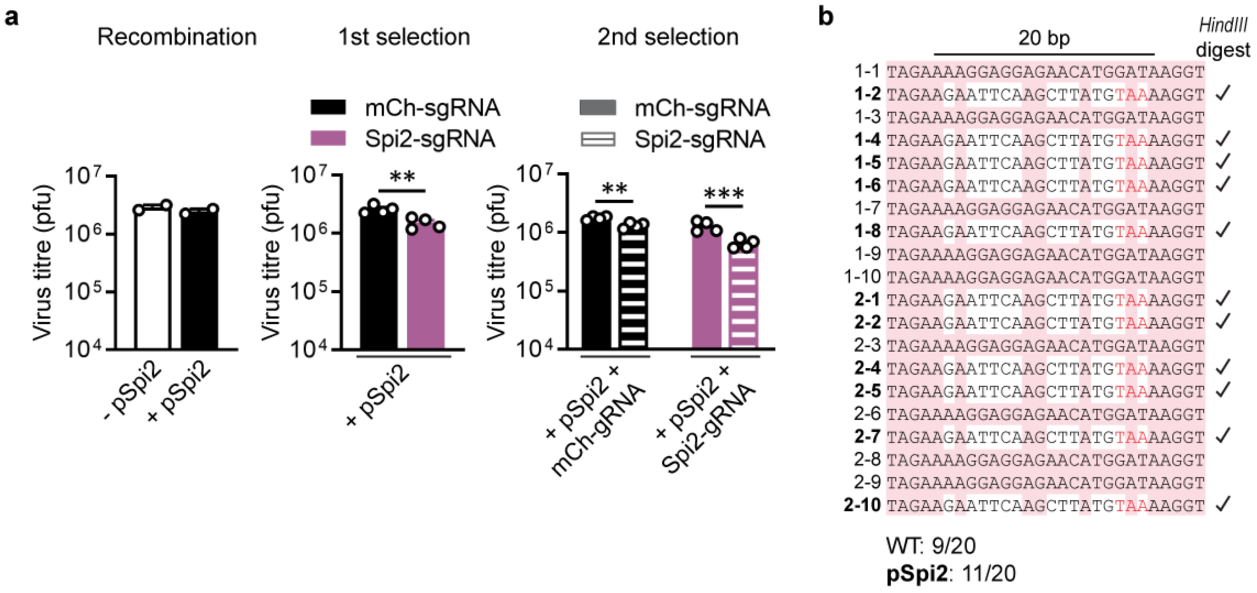

Supplementary Figure 5 – Marker-free recombinant VACV are accurately selected by Cas9 without evidence of additional mutation via NHEJ. a. Amounts of infectious virus from cultures shown in Figure 7b. Results are shown as means and SEM (** $p<0.01$, *** $p<0.001$ by Student's unpaired t-test (left and middle panel) and one-way ANOVA (right panel), n=2). **b.** Sequences at the recombination site of virus isolates from 20 plaques, 10 from each of two independent experiments. Sequences are marked to show the bases conserved with wild type VACV (highlighted in pink), the premature stop codon (red) and sequences matching desired knockout (bold labels). Number of sequences matching wild type VACV (WT) and desired knockout (pSpi2) are shown below. To the right shows isolates where PCR products spanning the recombination site can be cut with *HindIII*, as expected for desired recombinants.
