# Supplementary material for: Rapid poxvirus engineering using CRISPR/Cas9 as a selection tool
Source: Commun Biol. 2020 Nov 3;3:643. doi: 10.1038/s42003-020-01374-6 (PMC7641209; doi:10.1038/s42003-020-01374-6)
Supplement: Supplementary file 3 — Description of Additional Supplementary Files [file 42003_2020_1374_MOESM3_ESM.pdf]

Description of Additional Supplementary Files

File Name: Supplementary Video 1
Description: Cas9-mediated selection of recombinant VACVs in real-time. Live-cell imaging over 72 hours of Cas9-mediated selection of Venus+ virus produced by conventional recombination between VACV-mCh and the plasmid pSC11-Venus as described in Figure 5c. Imaging was performed using the IncuCyte S3 apparatus. Video shown is representative of three independent experiments are shown.

File Name: Supplementary Data 1
Description: Source data for this study.
