# Supplementary material for: Rapid poxvirus engineering using CRISPR/Cas9 as a selection tool
Source: Commun Biol. 2020 Nov 3;3:643. doi: 10.1038/s42003-020-01374-6 (PMC7641209; doi:10.1038/s42003-020-01374-6)
Supplement: Supplementary file 6 — Reporting Summary [file 42003_2020_1374_MOESM6_ESM.pdf]

nature research

Corresponding author(s): David C. Tscharke

Last updated by author(s): May 22, 2020

Reporting Summary

Nature Research wishes to improve the reproducibility of the work that we publish. This form provides structure for consistency and transparency in reporting. For further information on Nature Research policies, see our Editorial Policies and the Editorial Policy Checklist.

Statistics

For all statistical analyses, confirm that the following items are present in the figure legend, table legend, main text, or Methods section.

n/a	Confirmed	
☐	☒	The exact sample size (*n*) for each experimental group/condition, given as a discrete number and unit of measurement
☐	☒	A statement on whether measurements were taken from distinct samples or whether the same sample was measured repeatedly
☐	☒	The statistical test(s) used AND whether they are one- or two-sided<br>*Only common tests should be described solely by name; describe more complex techniques in the Methods section.*
☒	☐	A description of all covariates tested
☒	☐	A description of any assumptions or corrections, such as tests of normality and adjustment for multiple comparisons
☐	☒	A full description of the statistical parameters including central tendency (e.g. means) or other basic estimates (e.g. regression coefficient) AND variation (e.g. standard deviation) or associated estimates of uncertainty (e.g. confidence intervals)
☒	☐	For null hypothesis testing, the test statistic (e.g. *F*, *t*, *r*) with confidence intervals, effect sizes, degrees of freedom and *P* value noted<br>*Give P values as exact values whenever suitable.*
☒	☐	For Bayesian analysis, information on the choice of priors and Markov chain Monte Carlo settings
☒	☐	For hierarchical and complex designs, identification of the appropriate level for tests and full reporting of outcomes
☒	☐	Estimates of effect sizes (e.g. Cohen's *d*, Pearson's *r*), indicating how they were calculated

Our web collection on statistics for biologists contains articles on many of the points above.

Software and code

Policy information about availability of computer code

Data collection	Incucyte S3 software was used to collect live-cell imaging data and CellSens Entry was used for all other imaging. BD Accuri C6 Software was used to collect flow cytometry data.
Data analysis	ImageJ v1.53 was used to analyse immunofluorescence images and annotate video files. GraphPad Prism v8.4.2 was used for statistical analyses. FlowJo v10.6.2 was used to compile flow cytometry data.

For manuscripts utilizing custom algorithms or software that are central to the research but not yet described in published literature, software must be made available to editors and reviewers. We strongly encourage code deposition in a community repository (e.g. GitHub). See the Nature Research guidelines for submitting code & software for further information.

Data

Policy information about availability of data

All manuscripts must include a data availability statement. This statement should provide the following information, where applicable:

- Accession codes, unique identifiers, or web links for publicly available datasets
- A list of figures that have associated raw data
- A description of any restrictions on data availability

The data that support the findings of this study are available in this manuscript or from the corresponding author upon request. Source data for Figures 1b-e, g, 2b, 2d, 3a-h, 4a-4f, 5a, b, 6b and 7c and Supplementary Figures 2, 3 and 4 and 5a are provided with the paper.

April 2020

Field-specific reporting

Please select the one below that is the best fit for your research. If you are not sure, read the appropriate sections before making your selection.

☒ Life sciences ☐ Behavioural & social sciences ☐ Ecological, evolutionary & environmental sciences

For a reference copy of the document with all sections, see nature.com/documents/nr-reporting-summary-flat.pdf

Life sciences study design

All studies must disclose on these points even when the disclosure is negative.

Sample size	Sample size calculations were not performed on cell numbers, as cell lines consisted of clonal populations. For virus plaque analysis, 30-50 plaques are considered standard for analysis. We increased this number to 100 to gain a better insight into virus frequencies within mixed populations. For flow cytometry analysis, 25,000 cells was considered sufficient per sample. All other samples were analysed in their entirety.
Data exclusions	No data were excluded from our study.
Replication	All studies were replicated 2-3 times independently. Only data points representing independent experiments are shown, except for viral factory analyses in which the nature of the data points are described in the figure legend.
Randomization	Allocation was not randomized as all work was conducted with clonal cell lines.
Blinding	Blinding was not possible in this study due to the nature of transfection/infection experimentation.

Reporting for specific materials, systems and methods

We require information from authors about some types of materials, experimental systems and methods used in many studies. Here, indicate whether each material, system or method listed is relevant to your study. If you are not sure if a list item applies to your research, read the appropriate section before selecting a response.

Materials & experimental systems

n/a	Involved in the study	
☐	☒	Antibodies
☐	☒	Eukaryotic cell lines
☒	☐	Palaeontology and archaeology
☒	☐	Animals and other organisms
☒	☐	Human research participants
☒	☐	Clinical data
☒	☐	Dual use research of concern

Methods

n/a	Involved in the study	
☒	☐	ChIP-seq
☐	☒	Flow cytometry
☒	☐	MRI-based neuroimaging

Antibodies

Antibodies used	Anti-VACV antibody was generated within the laboratory using an established rabbit immunisation model
Validation	Antibody has been validated through publication for the detection of VACV in infected mammalian cells (DOI: 10.1016/j.virol.2014.03.020)

Eukaryotic cell lines

Policy information about cell lines

Cell line source(s)	293A cells were purchased in the US and BS-C-1 cells were a kind gift from J. Yewdell (NIH)
Authentication	None of the cell lines used were authenticated
Mycoplasma contamination	Cells tested negative for Mycoplasma in 2019
Commonly misidentified lines (See ICLAC register)	N/A

Flow Cytometry

Plots

Confirm that:

☒ The axis labels state the marker and fluorochrome used (e.g. CD4-FITC).

☒ The axis scales are clearly visible. Include numbers along axes only for bottom left plot of group (a 'group' is an analysis of identical markers).

☒ All plots are contour plots with outliers or pseudocolor plots.

☒ A numerical value for number of cells or percentage (with statistics) is provided.

Methodology

Sample preparation	Flow cytometry was conducted on cell scrapings following infection and transfection. Briefly, cells and supernatant from 6-well plates were collected and 250uL was taken for analysis. Cells were spun down in a 96-well plate, washed in PBS, fixed in paraformaldehyde and resuspended in FACS PBS for analysis. This was always done within 2 hours of collection and without freeze thaw cycles.
Instrument	BD Accuri C6 Cytometer was used to collect data.
Software	BD Accuri C6 Software was used to collect data.
Cell population abundance	A minimum of 25,000 cells were analysed for each sample, per experiment. Five independent experiments were conducted.
Gating strategy	Gating strategy was used to sort single cells, using an SSC/SSC plot followed by a FSC/FSC plot. No further cells were excluded by gating. Boundary of the negative population was determined using a non-fluorescent cell sample for each experiment.

☒ Tick this box to confirm that a figure exemplifying the gating strategy is provided in the Supplementary Information.
